# The AlpArray Seismic Network: A Large-Scale European Experiment to Image the Alpine Orogen

**DOI:** 10.1007/s10712-018-9472-4

**Published:** 2018-04-18

**Authors:** György Hetényi, Irene Molinari, John Clinton, Götz Bokelmann, István Bondár, Wayne C. Crawford, Jean-Xavier Dessa, Cécile Doubre, Wolfgang Friederich, Florian Fuchs, Domenico Giardini, Zoltán Gráczer, Mark R. Handy, Marijan Herak, Yan Jia, Edi Kissling, Heidrun Kopp, Michael Korn, Lucia Margheriti, Thomas Meier, Marco Mucciarelli, Anne Paul, Damiano Pesaresi, Claudia Piromallo, Thomas Plenefisch, Jaroslava Plomerová, Joachim Ritter, Georg Rümpker, Vesna Šipka, Daniele Spallarossa, Christine Thomas, Frederik Tilmann, Joachim Wassermann, Michael Weber, Zoltán Wéber, Viktor Wesztergom, Mladen Živčić, AlpArray Seismic Network Team, Rafael Abreu, Ivo Allegretti, Maria-Theresia Apoloner, Coralie Aubert, Simon Besançon, Maxime Bès de Berc, Didier Brunel, Marco Capello, Martina Čarman, Adriano Cavaliere, Jérôme Chèze, Claudio Chiarabba, Glenn Cougoulat, Luigia Cristiano, Tibor Czifra, Ezio D’Alema, Stefania Danesi, Romuald Daniel, Anke Dannowski, Iva Dasović, Anne Deschamps, Sven Egdorf, Tomislav Fiket, Kasper Fischer, Sigward Funke, Aladino Govoni, Gidera Gröschl, Stefan Heimers, Ben Heit, Davorka Herak, Johann Huber, Dejan Jarić, Petr Jedlička, Hélène Jund, Stefan Klingen, Bernhard Klotz, Petr Kolínský, Josef Kotek, Lothar Kühne, Krešo Kuk, Dietrich Lange, Jürgen Loos, Sara Lovati, Deny Malengros, Christophe Maron, Xavier Martin, Marco Massa, Francesco Mazzarini, Laurent Métral, Milena Moretti, Helena Munzarová, Anna Nardi, Jurij Pahor, Catherine Péquegnat, Florian Petersen, Davide Piccinini, Silvia Pondrelli, Snježan Prevolnik, Roman Racine, Marc Régnier, Miriam Reiss, Simone Salimbeni, Marco Santulin, Werner Scherer, Sven Schippkus, Detlef Schulte-Kortnack, Stefano Solarino, Kathrin Spieker, Josip Stipčević, Angelo Strollo, Bálint Süle, Gyöngyvér Szanyi, Eszter Szűcs, Martin Thorwart, Stefan Ueding, Massimiliano Vallocchia, Luděk Vecsey, René Voigt, Christian Weidle, Gauthier Weyland, Stefan Wiemer, Felix Wolf, David Wolyniec, Thomas Zieke, AlpArray OBS Cruise Crew, AlpArray Working Group

**Affiliations:** 1 Institute of Earth Sciences, Faculty of Geosciences and Environment, University of Lausanne, UNIL-Mouline Géopolis, 1015 Lausanne, Switzerland; 2 Institute of Geophysics, Department of Earth Sciences, ETH Zürich, Sonneggstrasse 5, 8092 Zurich, Switzerland; 3 Swiss Seismological Service at ETH Zürich, Sonneggstrasse 5, 8092 Zurich, Switzerland; 4 Geodetic and Geophysical Institute, Research Centre for Astronomy and Earth Sciences, Hungarian Academy of Sciences, Sopron, 9400 Hungary; 5 Department of Meteorology and Geophysics, University of Vienna, Althanstrasse 14, 1090 Vienna, Austria; 6 Kövesligethy Radó Seismological Observatory, MTA CSFK GGI, Meredek u. 18, Budapest, 1112 Hungary; 7 Institut de Physique du Globe de Paris, Sorbonne Paris Cité, Université Paris Diderot, UMR 7154 CNRS, 75238 Paris Cedex 05, France; 8 Université Côte d’Azur, UPMC, CNRS, Observatoire de la Côte d’Azur, IRD, Géoazur, 250 Rue Albert Einstein, 06560 Valbonne, France; 9 Institut de Physique du Globe de Strasbourg, UMR 7516, Université de Strasbourg / EOST, CNRS, 5 rue René Descartes, 67084 Strasbourg Cedex, France; 10 Institute of Geology, Mineralogy and Geophysics, Faculty of Geosciences, Ruhr-Universität Bochum, 44780 Bochum, Germany; 11 Freie Universität Berlin, Malteserstrasse 74-100, 12249 Berlin, Germany; 12 University of Zagreb, Horvatovac 95, 10 000 Zagreb, Croatia; 13 Zentralanstalt für Meteorologie und Geodynamik, Hohe Warte 38, 1190 Vienna, Austria; 14 GEOMAR Helmholtz Centre for Ocean Research Kiel, Wischhofstr. 1-3, 24148 Kiel, Germany; 15 Christian-Albrechts Universität Kiel, Otto-Hahn-Platz 1, 24118 Kiel, Germany; 16 University of Leipzig, Talstrasse 35, 04103 Leipzig, Germany; 17 Istituto Nazionale di Geofisica e Vulcanologia, Via di Vigna Murata 605, 00143 Rome, Italy; 18 Istituto Nazionale di Oceanografia e di Geofisica Sperimentale, Via Treviso 55, 33100 Udine, Italy; 19 Université Grenoble Alpes, Université Savoie Mont-Blanc, CNRS, IRD, IFSTTAR, ISTerre, 38000 Grenoble, France; 20 Bundesanstalt für Geowissenschaften und Rohstoffe, Geozentrum Hannover, Stilleweg 2, 30655 Hannover, Germany; 21 Institute of Geophysics, Czech Academy of Sciences, Boční II 1401/1a, 141 31 Prague 4, Czech Republic; 22 Geophysical Institute, Karlsruhe Institute of Technology KIT, Hertzstr. 16, 76187 Karlsruhe, Germany; 23 Institute of Geosciences, Goethe University Frankfurt, Altenhöferallee 1, 60438 Frankfurt am Main, Germany; 24 Republic Hydrometeorological Service of Republic of Srpska, 7800 Banja Luka, Bosnia and Herzegovina; 25 Dipartimento di Scienze della Terra dell’Ambiente e della Vita, Università degli Studi di Genova, Corso Europa, 26, 16132 Genoa, Italy; 26 Institut für Geophysik, Westfälische Wilhelms-Universität Münster, Corrensstrasse 24, 48149 Münster, Germany; 27 Helmholtzzentrum Potsdam, Deutsches GeoForschungsZentrum GFZ, Telegrafenberg, 14473 Potsdam, Germany; 28 Department of Earth and Environmental Sciences, Ludwig-Maximilians-Universität, Theresienstrasse 41, 80333 Munich, Germany; 29 Institute of Earth and Environmental Science, University of Potsdam, Karl-Liebknecht-Strasse 24-25, 14476 Potsdam-Golm, Germany; 30 Slovenian Environment Agency, Vojkova 1b, 1000 Ljubljana, Slovenia

**Keywords:** Seismology, Alps, Seismic network, Geodynamics, Seismic imaging, Mountain building

## Abstract

The AlpArray programme is a multinational, European consortium to advance our understanding of orogenesis and its relationship to mantle dynamics, plate reorganizations, surface processes and seismic hazard in the Alps–Apennines–Carpathians–Dinarides orogenic system. The AlpArray Seismic Network has been deployed with contributions from 36 institutions from 11 countries to map physical properties of the lithosphere and asthenosphere in 3D and thus to obtain new, high-resolution geophysical images of structures from the surface down to the base of the mantle transition zone. With over 600 broadband stations operated for 2 years, this seismic experiment is one of the largest simultaneously operated seismological networks in the academic domain, employing hexagonal coverage with station spacing at less than 52 km. This dense and regularly spaced experiment is made possible by the coordinated coeval deployment of temporary stations from numerous national pools, including ocean-bottom seismometers, which were funded by different national agencies. They combine with permanent networks, which also required the cooperation of many different operators. Together these stations ultimately fill coverage gaps. Following a short overview of previous large-scale seismological experiments in the Alpine region, we here present the goals, construction, deployment, characteristics and data management of the AlpArray Seismic Network, which will provide data that is expected to be unprecedented in quality to image the complex Alpine mountains at depth.

**Electronic supplementary material:**

The online version of this article (10.1007/s10712-018-9472-4) contains supplementary material, which is available to authorized users.

## Introduction

The long history of geological and geophysical research in Alpino-type European orogenic systems has revealed the nature of a complex and ever-varying orogenic system, its central segment being the Alps. The complexity mainly stems from the spatial heterogeneity of key lithospheric structures and their variation in time and space (e.g., Handy et al. 2010). In other orogens like the Himalaya or the Andes, structures in the crust or upper mantle can be traced continuously for hundreds, even thousands of kilometres (e.g., Oncken et al. 2006; Hetényi et al. 2016). In comparison, the European Alps including the adjacent Apennines, Dinarides and Carpathians show remarkable lateral (along-strike) variations in structure and style, such that their underlying processes are still subject to debates (see below). Many controversies regarding this complexity can be resolved by obtaining high-resolution images of the deep subsurface and by integrating these with surface studies supported by theoretical and modelling work. Past efforts employing seismic networks were insufficient to resolve orogen-scale targets and provide a physical basis for seismic hazard assessment in a comprehensive way. The AlpArray programme was therefore born as a multidisciplinary, research-oriented project to survey the greater Alpine area homogeneously with various geophysical methods.

The primary field focus has been on designing, deploying and operating a dense seismological network of broadband sensors. Experience over the past decade (e.g., within the USArray, IberArray) has shown that similar networks were very successful in imaging lithospheric and upper mantle structures (e.g., Moschetti et al. 2010; Levander et al. 2011). In the Alps, we therefore designed a target-oriented network at a spatial density that has been made possible only through the joint efforts of numerous participating institutions. Building on a growing network of permanent seismic stations, the AlpArray Seismic Network (AASN) was created by assembling sufficient number of temporary stations secured by funds from several countries. With over 600 stations deployed simultaneously there is rarely any place in the greater Alpine area that would be farther than 30 km from a broadband seismic station. Complementary ocean-bottom seismometers (OBS) were deployed in the Ligurian Sea. The resulting station distribution makes the AASN the ideal tool for 3-dimensional mapping of structures and physical properties of rock volumes in the crust, the upper mantle and the mantle transition zone.

In this paper we provide an overview of the evolution of AASN: its goals, construction principles, achieved characteristics, as well as complementary seismological experiments.

## Geodynamic Setting, Questions and Goals

**Fig. 1:**
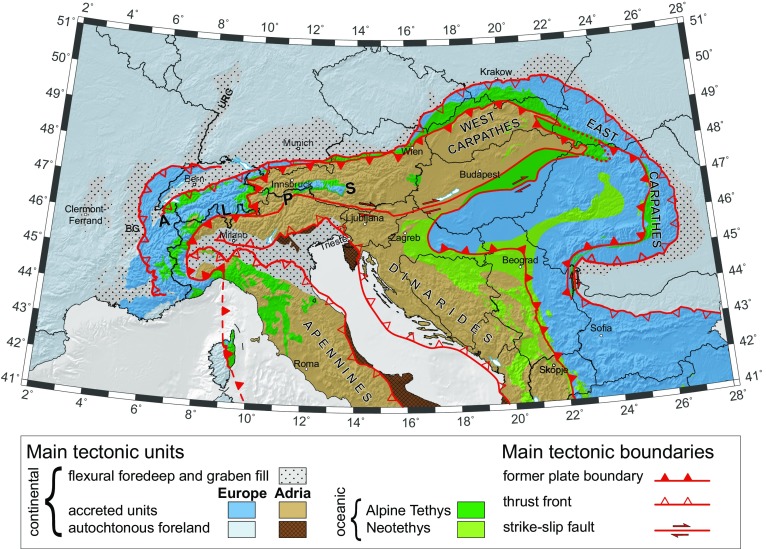
Simplified tectonic map of the Alps, their adjacent orogens and forelands that result from the collision of two converging plates, Europe and Adria. The major units are coloured according to their plate-tectonic provenance. Red colours mark the main tectonic boundaries related to plate-kinematic history of the Alps. *URG* Upper Rhine Graben. *BG* Bresse Graben. Modified from Schmid et al. (2008), Ustaszewski et al. (2008), Schmid and Slejko (2009), Handy et al. (2010) and Bousquet et al. (2012)

The greater Alpine tectonic area (Fig. 1) comprises an orogenic system formed by the interaction of the Adriatic and European plates. The Adriatic plate is one of several microplates of oceanic and continental provenance whose interaction between two large converging plates, Europe and Africa, has given rise to the highly arcuate Alpine–Mediterranean mountain belt (e.g., Handy et al. 2010). This arcuation reflects different episodes of slab retreat since 85–90 Ma in the Alps, Apennines and Carpathians. Geological studies have long shown that while the Adriatic plate is the upper plate in the Western and Central Alps and the Western Carpathians, it forms the lower plate in the Apennines and the Dinarides (e.g., Schmid et al. 2008; Handy et al. 2015). This complex geodynamic setting is reflected in the Moho morphology (e.g., Brückl et al. 2010; Di Stefano et al. 2011; Spada et al. 2013) and in the seismic properties of the crust and the mantle. Travel-time ambient-noise seismic tomography has imaged 3D seismic velocity anomalies in the crust (e.g., Diehl et al. 2009; Di Stefano et al. 2009, 2011; Molinari et al. 2015) and the upper mantle (e.g., Babuška et al. 1990; Lippitsch et al. 2003; Piromallo and Morelli 2003; Spakman and Wortel 2004; Kissling et al. 2006; Mitterbauer et al. 2011; Giacomuzzi et al. 2011, 2012; Zhao et al. 2016), leading to often controversial geodynamic interpretations. While most authors agree that mantle slabs are spatially linked to both modern (at shallow depth) and ancient (at greater depth) zones of lithospheric subduction, considerable uncertainties about slab geometry, internal properties, tears and even orientation remain. At the Apennines–Alps transition zone, existing data do not allow us to discern the geometry of the switch in slab polarity, which is so far only gleaned from nappe vergence at the surface (e.g., Faccenna et al. 2001; Piromallo and Faccenna 2004; Vignaroli et al. 2008, 2009; Handy et al. 2010; Schmid et al. 2017). Beneath the Eastern Alps, the exact outlines and origin of a slab anomaly are the subject of ongoing controversy between proponents of a presently N-dipping Adriatic subduction (Lippitsch et al. 2003; Schmid et al. 2004; Kissling et al. 2006; Handy et al. 2015; Zhao et al. 2016) versus advocates of European subduction (Mitterbauer et al. 2011), complemented with suggestions on a dual origin of the slab (Babuška et al. 1990). Solving this controversy is particularly relevant for the Friuli seismic hazard area, which is located either in the foreland (if the slab beneath the Eastern Alps is Adriatic) or in the hinterland of the Alps (if the slab beneath the Eastern Alps remained European). In general for the entire region, the determination of mantle seismic anisotropy patterns helps to reconstruct the current and past plate motions and dynamics in three dimensions; the broad coverage with the uniform AASN will allow a more comprehensive analysis with respect to previous studies often focused on smaller areas (Margheriti et al. 2003; Plomerová et al. 2006; Kummerow et al. 2006; Fry et al. 2010; Barruol et al. 2011; Salimbeni et al. 2013; Qorbani et al. 2016; Subašić et al. 2017).

These and other Alpine debates raise the question of data resolution and coverage and call for an integrated geophysical–geological approach. Are some of the published models’ inconsistencies due to sparse sampling and to (overlooked) artefacts? Can they be overcome by optimizing seismic network aperture and station coverage? The purely passive experiments planned by AlpArray involve a more densely spaced network than previous experiments. They provide an excellent opportunity to extend our knowledge of lower crustal structure and the crust–mantle interface (the Moho), beyond current models based on the controlled-source seismology of the last century. Furthermore, AlpArray will allow imaging of structural details and fabric of the mantle lithosphere and the lithosphere–asthenosphere boundary (e.g., Plomerová and Babuška 2010) that are currently rather poorly constrained beneath the orogens compared to some parts of the foreland (e.g., Plomerová et al. 2012). Better-constrained information on the deep structure of the greater Alpine area will in turn offer fresh insight into different stages of mountain building and allow us to study how subduction and collision in the Alps have interfered with neighbouring rift structures (Rhine–Bresse system, Fig. 1).

The societal relevance of the AlpArray project clearly lies in the opportunity to improve seismic hazard maps (e.g., Stucchi et al. 2012; Grünthal et al. 2013). This is especially needed for areas that are among the most densely populated in Europe: while the Alps sensu stricto host “only” 14 million people in ca. 0.2 million km^2^, the greater Alpine area (as defined in Sect. 4.1) is the home of about 115 million people (CIESIN and CIAT 2005) and a significant economic value over ca. 0.9 million km^2^. The homogeneous data provided by AlpArray will augment the reliability of earthquake hazard assessments.

The Alps are one of the (if not “the”) best-studied active mountain ranges in the world. They have exceptional geophysical and geological databases which are excellent requisites for advancing our general knowledge and understanding of orogenic processes on all scales. The scientific controversies and questions above make it evident that the time is ripe for a new generation of cross-disciplinary geophysical–geodynamical initiatives. These will shed new light on the current state and long-term evolution of the deep structure of the Alps at crustal and mantle depths. The AlpArray Seismic Network is designed as the main field component of the AlpArray programme and will hopefully provide high-resolution, large-aperture seismological data for unprecedented views of orogenesis.

## Historical Perspective

### Seismological Observations in the Alps

#### Observatory Versus Research Campaign

The seismological investigation of the European Alps developed essentially along two tracks. On the one hand, national or regional observatories, mandated to monitor seismicity, targeted strategic positions with permanent sensors to build various seismic networks serving global, regional or local purposes. In general, these sites are carefully prepared in a time-intensive manner, with infrastructure that is appropriate for broadband, short-period or accelerometric sensors. On the other hand, research institutions usually took occasional initiatives to carry out temporary campaigns, with targeted ideas about the network geometry, operation duration and other characteristics. Because of their temporary nature — and sometimes the need for building numerous sites within a short time — these sites are generally selected and installed more quickly than permanent ones with a consequence of lower signal-to-noise measuring conditions. As a result, observatory and research targets generally do not coincide. Further, it has been challenging to realize the integrated curation of data sets. While much was learned about particular aspects of the Alpine convergence, these separate initiatives typically lacked the critical mass to resolve the larger-scale structure in a coherent manner such that even many first-order questions such as the one on the polarity of the subduction below parts of the Alps remained unresolved.

#### Brief History of Seismological Investigations

The early history of devices able to record seismic motions is difficult to trace, but they existed in Europe at the latest by the early eighteenth century (Ferrari 1992). Seismometers targeting long-period motions began being installed across the Alps around the turn of the nineteenth–twentieth century, e.g., in 1897 in Ljubljana, or in 1911 at Degenried, Zurich, at a site that is still occupied today (CH.ZUR). In the mid-1980s digital seismometry began in earnest, and increased disc capacities made the operation of continuously recording temporary stations a possibility, and greatly simplified the operation of permanent stations. Within the AlpArray area, the earliest permanent stations that are still in operation today were installed or upgraded to digital in the 1980s and early 1990s. Fortunately, by the time of inception of the AlpArray project, many countries in the Alpine region operated dense and mature permanent broadband seismic networks.

**Fig. 2:**
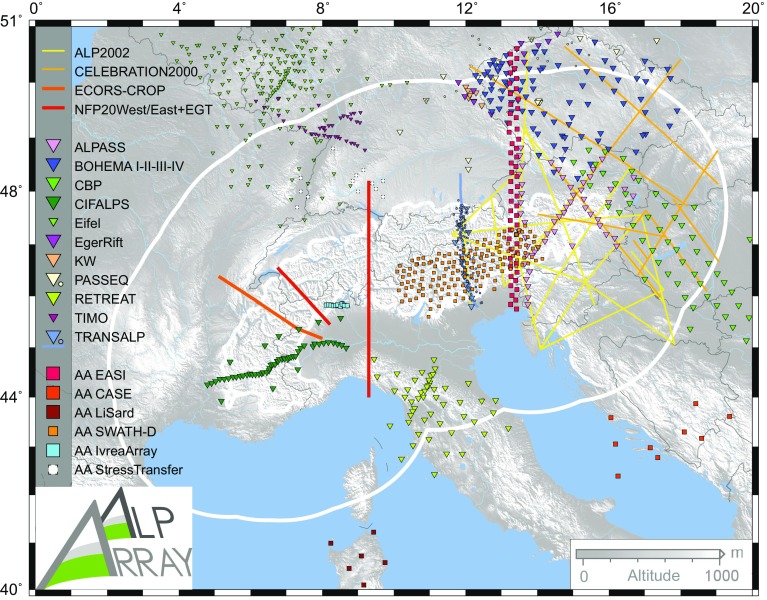
Location of selected seismological project in the greater Alpine area. Active seismic campaigns are shown as solid lines. Past passive seismological networks are shown as symbols (broadband: large triangles, short period: small circles, mixed: small triangles). AlpArray complementary experiments are shown as squares (past and current: solid contour, planned: dashed contour). See text for overview

Most of our knowledge of the Alpine subsurface (especially the crust) comes from active, controlled-source seismic campaigns (CSS) (Fig. 2). These CSS provide higher-resolution images of the crust than the currently popular passive methods (e.g., local earthquake tomography, receiver functions), but their limited depth penetration, permitting procedure (explosive or vibration source) and especially cost limit frequent use in Alpine research as compared to the past. The list of active-source experiments and experiment series is long and covers the continent, for an overview we refer to Prodehl and Mooney (2012). Let us here remember the earliest active seismic observations (Reich 1952), the campaigns carried out between 1956 and 1960 in the Western Alps (Closs and Labrouste 1963) and in the 1960s in the Bohemian Massif and the Western Carpathians (see Beránek et al. 1975 for review). These were followed by several others (e.g., ALP75: Alpine Explosion Seismology Group 1976; ECORS-CROP: Roure et al. 1990; European Geotraverse EGT: Blundell et al. 1992; NFP20: Pfiffner et al. 1997; GéoFrance 3D: GéoFrance 3D (1999); TRANSALP: Kummerow et al. 2004; Alp2002: Brückl et al. 2003; CELEBRATION2000, e.g., Grad et al. 2006; see Fig. 2 for some locations), some of which were also accompanied by temporary passive seismic deployments.

To our knowledge broadband campaigns in the greater Alpine region started in 1992, by a small-size passive experiment in Central Europe in which the Gräfenberg array was extended to the North. The experiment aimed at collecting data for a more detailed study of the Saxothuringian-Moldanubian lithosphere unit contact (Plomerová et al. 1998). Subsequent campaigns (see location examples in Fig. 2) often aimed at crustal, lithospheric and upper mantle depths by various passive seismology methods, all along the Alpine arc from the West (e.g., French Massif Central: Granet et al. 1995; CIFALPS: Zhao et al. 2015) and Northwest (EIFEL: Ritter et al. 2000; TIMO: Ritter et al. 2009), through the Central Alps (ALPASS: Brückl et al. 2005; TRANSALP: Kummerow et al., 2004) to the Eastern termination (e.g., CBP project: Hetényi et al. 2009, 2015; Dando et al. 2011; Ren et al. 2013), in the Apennines to the South of the Alps (GeoModAp: Amato et al. 1998; RETREAT, e.g., Plomerová et al. 2006; Margheriti et al. 2006; Munzarová et al. 2013) and in the Bohemian Massif adjacent to the Alps on its northern side (MOSAIC: Plomerová et al. 2005; BOHEMA, e.g., Karousová et al. 2013; PASSEQ: Wilde-Piórko et al. 2008; Eger Rift, e.g. Geissler et al. 2005; KW: Bianchi et al. 2015).

While all these efforts have headed towards the first major experiment with significant aperture and dense spatial coverage, i.e. the AlpArray Seismic Network, the AlpArray programme also mobilizes remaining energies, funds and broadband instruments in Europe to carry out a number of targeted AlpArray Complementary Experiments. These projects are (Fig. 2):

- EASI (for Eastern Alpine Seismic Investigation) with 55 stations in a N-S transect along 13.3°E (AlpArray Seismic Network 2014);
- CASE (for Central Adriatic Seismic Experiment) filling the coverage gap as much as possible across the Adriatic Sea (AlpArray Seismic Network 2016);
- LiSard (The Lithosphere of Sardinia) to broaden the aperture of the AASN (http://host.uniroma3.it/progetti/lisard/project.html);
- IvreaArray, a 10-station E–W-oriented array of 5-km spacing to focus on a section of the geophysical Ivrea Body (Hetényi et al. 2017), where an ICDP initiative aims to drill down to several kilometres depth (project DIVE: Drilling the Ivrea-Verbano zonE; Pistone et al. 2017);
- SWATH-D, an initiative that further increases the effective station density of the AASN along an E–W corridor in the southern Central and Eastern Alps (Heit et al. 2017), and straddles the hypothesized polarity change of the subduction and the lower crustal suture zones;
- StressTransfer, a project to study stress transfer and Quaternary faulting in the northern Alpine foreland.

Furthermore, the ocean bottom seismometer (OBS) component of AlpArray in the Ligurian Sea has triggered a further three marine or amphibious experiments, planned to be finalized in 2018:

- LOBSTER (Ligurian Ocean Bottom Seismology and Tectonics Research), an onshore–offshore refraction experiment with 3 land stations and 35 short-period OBSs, which has been performed in February 2018 along two profiles. The additional benefit is that AlpArray OBSs will also record these active sources which will allow to retrieve proper station orientation information;
- OBS-Ligure, a local densification of the AlpArray Seismic Network with 7 short-period OBSs at the Northern Ligurian margin, in an effort to better record the seismicity in a region where faults are known to be capable of generating rare earthquakes in a range of *M*w 6–7 (e.g. Larroque et al. 2012);
- SEFASILS, featuring 50–60 OBSs and a dozen onshore sites for refraction and deep reflection studies between Corsica and the toe of the continental slope between Nice and the Gulf of Genoa.

### Seismology with Large Networks and Arrays

Although there is no strict definition of a seismological array (Rost and Thomas 2002), one can distinguish two main classes (see below for examples, and Rost and Thomas 2002 for further examples).

Permanent, usually small aperture arrays mainly target the enhancement of weak signals, generally from distant sources, and record waveforms that are coherent across the array or between neighbouring stations for at least some period of interest. Irregular station spacings are often employed to achieve good array response over a range of frequencies.

Temporary, usually large-aperture networks are installed locally, in the area of interest, and generally feature regular station spacing to achieve uniform coverage of the underlying target. This design is optimized for tomographic and discontinuity imaging.

#### Permanent Arrays

The benefits of array seismology came from smaller but permanent arrays. The LASA (large-aperture seismic array, USA, e.g., Frosch and Green 1966) and NORSAR (Bungum et al. 1971) arrays both deployed over 100 stations on a permanent basis, but due to problems with noise and signal coherency the former was closed; the latter was reduced in size. Outstanding examples are the Yellowknife Array, with excellent location and noise conditions (e.g., Anglin 1971) and other UKAEA arrays (Keen et al. 1965). Close to the Alps the Gräfenberg Array (e.g., Buttkus 1986) also serves seismic observation purposes since 1976. Many other permanent arrays exist but cannot all be mentioned here.

#### Temporary Arrays or Networks

The number of temporary broadband campaigns has dramatically increased over the past two decades, but the number of large-aperture, high-density arrays is still small because of the huge logistical and coordination effort needed to construct one. During the past two decades, several large-scale arrays have been realized and operated in international cooperation in northern Europe, such as SVEKALAPKO (e.g., Hyvönen et al. 2000), TOR (e.g., TOR Working Group et al. 2002) and POLENET/LAPNET (e.g., Kozlovskaya and POLENET/LAPNET working group 2008). Here we highlight two large precursor projects for AlpArray with large aerial extent and near-regular station spacing.

The Transportable Array component of USArray (Meltzer et al. 1999; http://www.usarray.org), which is part of the EarthScope experiment, is a 15-year programme to place a dense network of permanent and portable seismographs across the continental USA on a rolling basis. Four hundred sensors are located on a 70-km spacing grid for more than 1.5 years during each campaign, to cover the contiguous 48 states and now Alaska. The funding by the US National Science Foundation included all the costs of infrastructures, including a new pool of mobile broadband seismic instruments, the costs of their deployment and operation in the field by a dedicated team, and new facilities for array operations, data collection, quality control and distribution by the IRIS Data Management Center. As a consequence, the collected data were directly and publicly open for use by researchers (IRIS Transportable Array 2003). USArray is certainly an example to follow for AlpArray; however, the political and funding conditions are neither comparable nor reachable in Europe, which necessarily have led to a very different organization (see below). Note that while the Transportable Array can also be used to study sources, it is generally employed as a large-aperture network.

IberArray (http://iberarray.ictja.csic.es) is a part of the TopoIberia programme and focused on and around the Iberian Peninsula. More than 50 seismometers have been deployed at a time in different phases moving northward from Morocco to the Pyrenees between 2007 and 2014. The over 200 sites have an average spacing of 60 km. The data became publicly available 3 years after the end of the experiment (Institute Earth Sciences “Jaume Almera” CSIC (ICTJA Spain) 2007). Many other temporary arrays have been deployed in the last decades, and their impact on understanding the Earth has been described in many publications that cannot all be mentioned here.

#### Further Array Plans

Array seismology is also on the rise in other regions to focus on well-defined geographic regions. As of the time of writing, the authors are aware of the following ongoing initiatives:

- CCArray: focusing on the Canadian Cordillera, planned (not necessarily simultaneous) operation of 200–400 land station and ca. 32 OBS stations, as well as 100–200 GPS stations, over 3 or more years
- ChinArray: focusing on mainland China, an experiment with several hundred stations on a rolling basis;
- AusArray: targeting Australia on a rolling basis, each phase is planned for at least 1-year periods at 55-km station spacing.

## AlpArray Seismic Network

### Network Geometry Construction

**Fig. 3:**
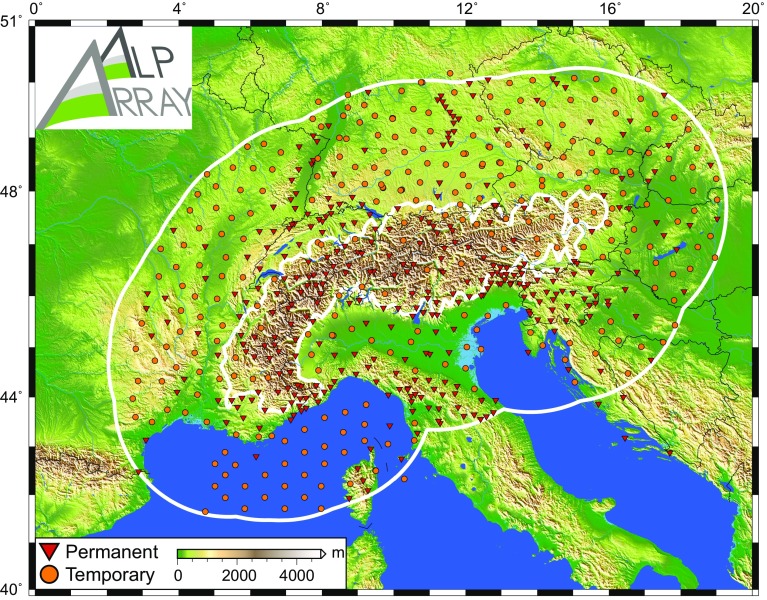
Topographical map of the greater Alpine area and the geometry of the AlpArray Seismic Network. The permanent and temporary broadband stations (respectively red triangles and orange circles) cover the area within 250 km of the smoothed 800-m altitude line of the Alps (outer and inner thick white lines). Status as of August 2017

To fulfil the goal of imaging the Alps and their neighbouring mountain belts down to the bottom of the mantle transition zone (ca. 660 km depth), the AASN must have sufficient aperture around the topographic expression of the Alps themselves. To delineate this “greater Alpine area” quantitatively, the targeted coverage zone of the AASN is considered to be less than 250 kilometres away from the smoothed 800-metre altitude contour around the Alps (Fig. 3).

The AASN’s skeleton is the network of permanent stations operated in the area with (1) three-component recordings (vertical and typically N-S and E-W), (2) broadband sensors at least up to 30-s period responses and (3) open access data with a sampling rate of at least 100 samples per second. This background network was an ever-moving target during the 6 years of AASN planning, as new stations were installed, existing ones closed, and some stations were made open, resulting in a ca. 50% increase from 234 stations in summer 2011 to 352 in summer 2017. This growth rate is beyond the typical evolution of mature permanent networks. The AlpArray initiative coincided with the development of the European Integrated Data Archive (EIDA), which encouraged open access to broadband seismic data sets from permanent networks across Europe. Nevertheless, the AlpArray initiative created additional momentum to encourage the opening of data sets and the construction of new stations in the Alpine area, thus creating a legacy beyond the lifetime of the ongoing temporary experiment.

The design target for the sites of the AASN temporary network was to obtain homogeneously spaced coverage with the minimum number of stations, while exploiting the existing permanent stations as well as possible. Thanks to the aforementioned progress in the permanent networks during the design phase, the daunting task of manually planning the location of temporary sites to best fill the gaps had to be repeated several times. The rewarding result was that not only could the several participants agree on the common plan of deployment, but also that the number of planned sites could be matched with the number of available instruments. Furthermore, agreements were rapidly made to share seismic instruments and manpower between project partners in order to achieve the desired coverage.

**Fig. 4:**
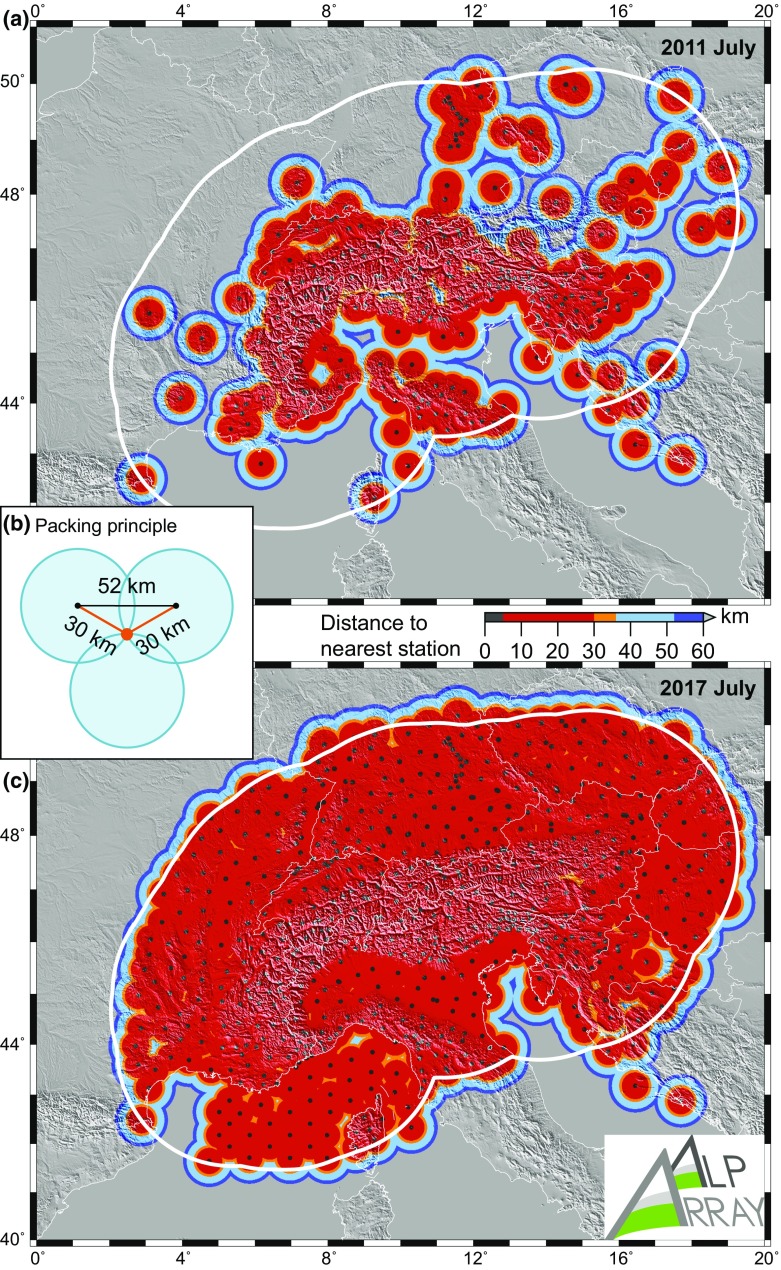
Map showing distance to the closest broadband seismological station: **a** spacing at the beginning of AlpArray planning in summer 2011 with 234 stations; **b** principle of positioning newly installed temporary stations; **c** spacing of the complete 628 stations of the AlpArray Seismic Network in August 2017

The final strategy for locating temporary sites within AASN was to adopt a hexagonal compact packing strategy (Fig. 4b). Instead of a rectangular grid, this geometry could better adapt to the existing permanent network stations and ensure that “voids” which are easily created in a grid are filled. Furthermore, only a 3-km radius area around each planned site location was allowed for station installation by the deploying teams. In case of larger deviation from a planned site, the neighbouring site locations were at least reconsidered. With this procedure, no point within the targeted area is farther than 30 km away from an AASN station. In newly covered regions each AASN station is at 52 km distance from 6 neighbouring sites, which is tighter and more compact than previous large networks around the world (see above).

Despite the thorough planning on land, three gaps remained within the targeted zone: (1) the largest one is naturally in the Ligurian Sea and the neighbouring Gulf of Lions, which we aimed to cover with ocean-bottom seismometers right at the start of the project conception. Following varying plans and station availabilities, the final configuration covers most of the Ligurian Sea, only leaving empty areas with less than 1000 m water depth, which cannot be instrumented as they are prone to bottom trawling. (2) The second major gap is in the Adriatic Sea, whose northern part is also too shallow for secure OBS operations. To minimize this gap, we planned the sites on land as close as possible to the coast. Furthermore, the CASE Complementary Experiment aims at closing the gap on the SE edge of the AASN. (3) In the SE corner of the AASN, gaps in the Dinarides were unavoidable, because stations could not be deployed safely in some areas. The AASN planning was optimized in this area following the local boundary conditions.

### Installation Preparation

The technical planning of the tremendous AASN operation was initiated as early as the scientific planning. A working group with various backgrounds (observatories, research institutes, network operators, mobile station pools, field experts, IT experts, etc.) was formed and developed a Technical Strategy for the AASN. This document sets “compulsory”, “best practice” and “recommended” rules in numerous aspects of the AASN installation and operation, such as equipment and settings; site selection, vault types and noise levels; communications and maintenance schedule; data recovery and security; data formats, access and coordination. This document was adopted as the standard for the operation of the AASN early on and was closely followed throughout the years. For further details, we refer to the document, available on the AlpArray website (http://www.alparray.ethz.ch/export/sites/alparray/.galleries/dwn-experiments/AlpArray_TechnicalStrategy.pdf).

Prior to the overall deployment a station naming convention was also adopted following the Standard for the Exchange of Earthquake Data (SEED) (IRIS 2012). A unique station name is composed of 5 alphanumeric characters: a leading “A” (for AlpArray), followed by three digits from a range of numbers distributed for each country, ending with an “A” for the initial site and changed to subsequent letters (B, C, etc.) in case of major (> 10 m) site changes. In this way the deploying groups could work in parallel and independently. The ranges of station names for the ten countries of temporary station deployment as well as the OBS component are shown in Table 1.

**Table 1:** Range of numbers for the middle 3 digits of AASN temporary station names

	Country of deployment	Range of numbers
AT	Austria	001–049
BH	Bosnia–Herzegovina	050–059
CH	Switzerland	060–069
CZ	Czech Republic	070–099
DE	Germany	100–149 and 350–399
FR	France	150–249
HR	Croatia	250–259
HU	Hungary	260–279
IT	Italy	280–329
SK	Slovakia	330–349
OBS	OBS component	400–449

AlpArray temporary stations belong to the Z3 network, a code reserved with the International Federation of Digital Seismograph Networks (FDSN).

Stations of permanent networks were generally not altered, neither in their names nor following the rules of the AlpArray Technical Strategy. However, to simplify data access, the entire AASN network data (both permanent and temporary stations) are available under a unique virtual network code “_ALPARRAY” (see Sect. 4.6).

### Installation

**Fig. 5:**
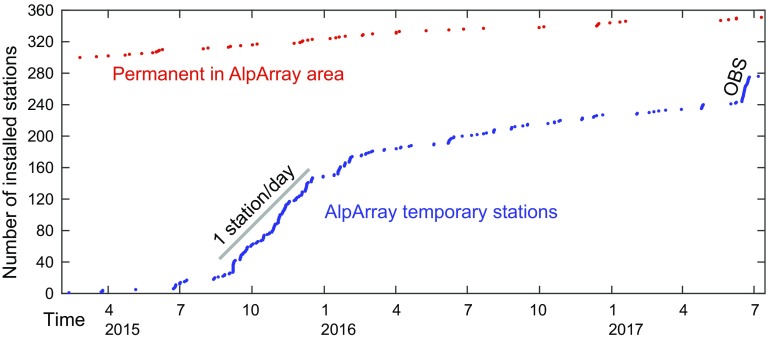
Development of AASN broadband seismological stations in the AlpArray area. Each point represents one station. Time is in month (above) and year (below) units. During the peak of the installation phase in autumn 2015, more than 1 station per day was installed. Only the ocean-bottom sensor (OBS) deployment rate was higher

The official start date of the AASN was 1 January 2016. Installation of the temporary stations had already started in summer 2015 and reached more than 81% on land by mid-2016 and more than 92% by the end of 2016 (Fig. 5). The AASN was fully completed in early July 2017, with the deployment of the OBS component and of the last land station. The main cause for this extended schedule was the heterogeneous funding scheme and logistic boundary conditions across the participating countries. The evolution of the AASN installation is shown in an animation provided as Online Resource 1.

We strived to keep common categories regarding housing and soil types of the installed stations. The various housing classes and the number of corresponding sites are listed in Table 2, along with the distribution of soil types on which the sensors sit. Regarding real-time communication of the stations, about 75% of the land stations could be equipped with an online communication device (Table 2). Among the permanent sites all except two stations are online. Full station details with photographs and further notes are kept up to date on the European Station Book hosted by the Orfeus Data Centre (http://www.orfeus-eu.org/opencms/stationbook/index.html).

**Table 2:** Housing classes, soil types and communication modes of temporary AASN sites (including already moved sites)

Housing class	Number of stations
Cave	2
Tunnel	3
Borehole	1
Underground shelter	19
Free field	53
Urban free field	47
Building	134
Other	8
OBS	30

Soil types	Number of stations
Bedrock or rock	7
Cement	199
Tiles	25
Tiles on cement	21
Cement on/in soil	5
Stone tiles	2
Other	8
Sea bottom	30

Communications	Number of stations
Online	184
Offline	63
OBS	30

*OBS* ocean-bottom seismometer

The completion of the AASN was achieved by a joint French–German OBS deployment cruise campaign in June 2017. The 30 stations deployed in the Ligurian Sea were offline by nature. Following a joint German–French OBS cruise in February 2018, 27 stations have been successfully recovered, 1 has been released from the ocean-bottom but not yet found, 1 could not be released but includes an automatic trigger set for October 2018. The 30th OBS was successfully recovered mid-March 2018 by a French cruise. Orientations of the horizontal components will be known after initial processing of the data.

### Challenges

The principal challenge in establishing the AASN was to achieve a coherent schedule. The main reason for this was the lack of unified European funding on par with that which enabled USArray or IberArray. Instead, funding to run AlpArray came from national sources (Austria, Croatia, Czech Republic, France, Germany, Hungary, Switzerland) complemented by institutional internal funding sources (Italy). Ultimately, all these initiatives were successful, but in some countries several re-submissions were required, such that the project durations in different countries did not always overlap, causing coordination headaches to ensure the design goal of all stations being operational at the same time. This was exacerbated by the complexity of instrument availability in numerous mobile pools, including the purchase of new hardware (new stations in Germany, France, Hungary). Therefore, the multivariate equation system comprising politics and logistics was resolved iteratively and required both cooperation and pragmatism over the years to result in the AASN as described here.

The successful cooperation between many partners called for internal rules and guidelines, which were not straightforward to realize. The AlpArray project has its own Memorandum of Collaboration for the overarching science programme (http://www.alparray.ethz.ch/export/sites/alparray/.galleries/dwn-experiments/MemorandumOfCollaboration_AlpArrayScientificProgram_150518.pdf). The AlpArray Seismic Network has developed rules for the participating institutions (http://www.alparray.ethz.ch/export/sites/alparray/.galleries/dwn-experiments/MemorandumOfCollaboration_AlpArraySeismicNetwork_150518.pdf); for example, an institute needs to operate at least ten stations to become a full member. Finding acceptable and respected rules for both observatories and universities, and for parties with different levels of access to new data, was a challenge, but also a prerequisite to beginning deployment and field operation.

Although the technical standards were clearly set from the beginning, it was not practical for some sites to meet certain recommendations, particularly with regard to background noise levels. An example are sites in the Po Plain, where the background noise level is very high, and finding an optimal site that meets the agreed noise level targets and also fitting the overall network geometry was impossible such that either the geometry or (in most cases) the noise criteria had to be relaxed. In other regions, several sites have not met the agreed noise criteria and have already been moved, which requires careful monitoring and responsive field teams.

Funding the OBS component with matching cruise schedules in Germany and France was an additional task, followed by further challenges regarding permits and ship routes, including last minute changes due to intervention by national navies.

### The Completed AASN

The AASN is composed of 628 stations in total: 352 permanent and 276 temporary seismometers, the latter including 30 ocean-bottom sensors. By August 2017 already 20 temporary stations had to be moved to occupy more favourable sites nearby, or because of changes in permitting situation. Full details of permanent and temporary stations, together with a GoogleEarth file, are provided as Online Resources 2, 3 and 4.

**Fig. 6:**
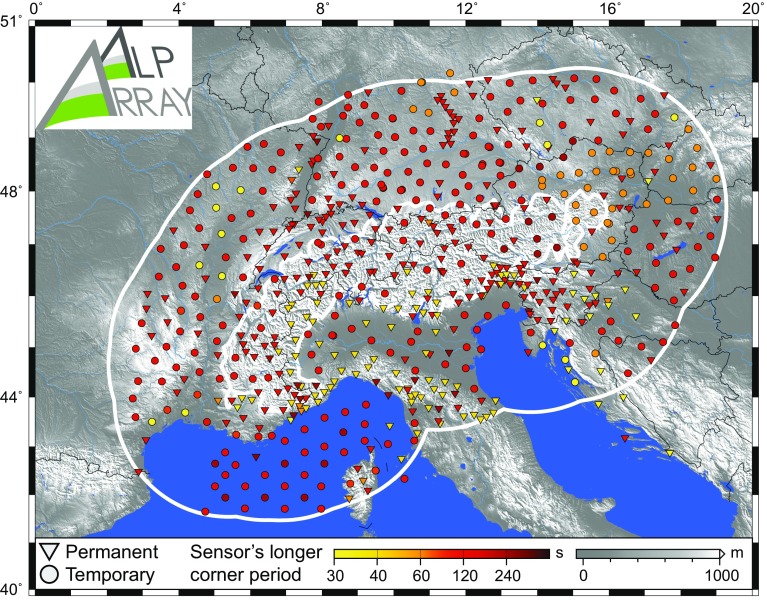
AlpArray Seismic Network “broadbandness”: the distribution of the longer corner period of seismological sensors. The great majority of sensors record beyond 100-s period. Status as of August 2017

The broadband character of AASN is shown in Fig. 6. While the minimum requirement was set to 30 s for the sensor’s upper corner period, the overwhelming majority (ca. 73%) of sensors have corner periods at 120 s or beyond.

**Fig. 7:**
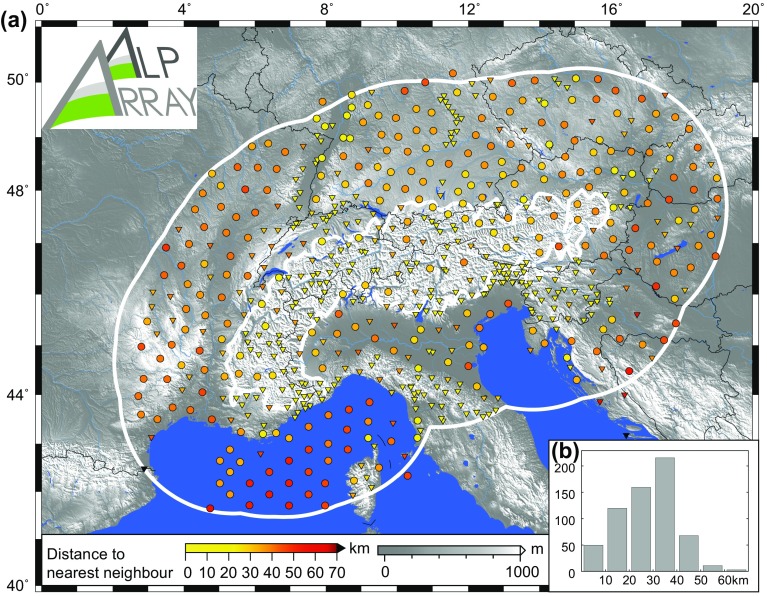
AlpArray Seismic Network vicinity: **a** map of distance to the nearest other seismological station; **b** histogram of these distances. As on previous figures, triangles denote permanent stations and circles represent temporary ones. Status as of August 2017

The success in achieving the targeted coverage is illustrated in Fig. 4, where red colour shows areas satisfying our spacing aims. The spatial density of the coverage is also represented by the station-wise map of distance to the nearest neighbouring station (Fig. 7).

The AASN is expected to operate until the end of 2018 at least, to ensure simultaneous data acquisition at land stations over 2 years. The recording period is obviously shorter for the OBS component (ca. 8 months) due to their autonomy related to current battery technology, as well as to the schedule of research vessels.

The AASN goes beyond previous large-scale initiatives in offering a higher station density and not following a rolling site occupation but simultaneous operation of all stations for a minimum period of 2 years. It is a measure of its success that it focuses on a geological target rather than being governed by political boundaries.

### Data Examples, Access and Network Perspectives

**Fig. 8:**
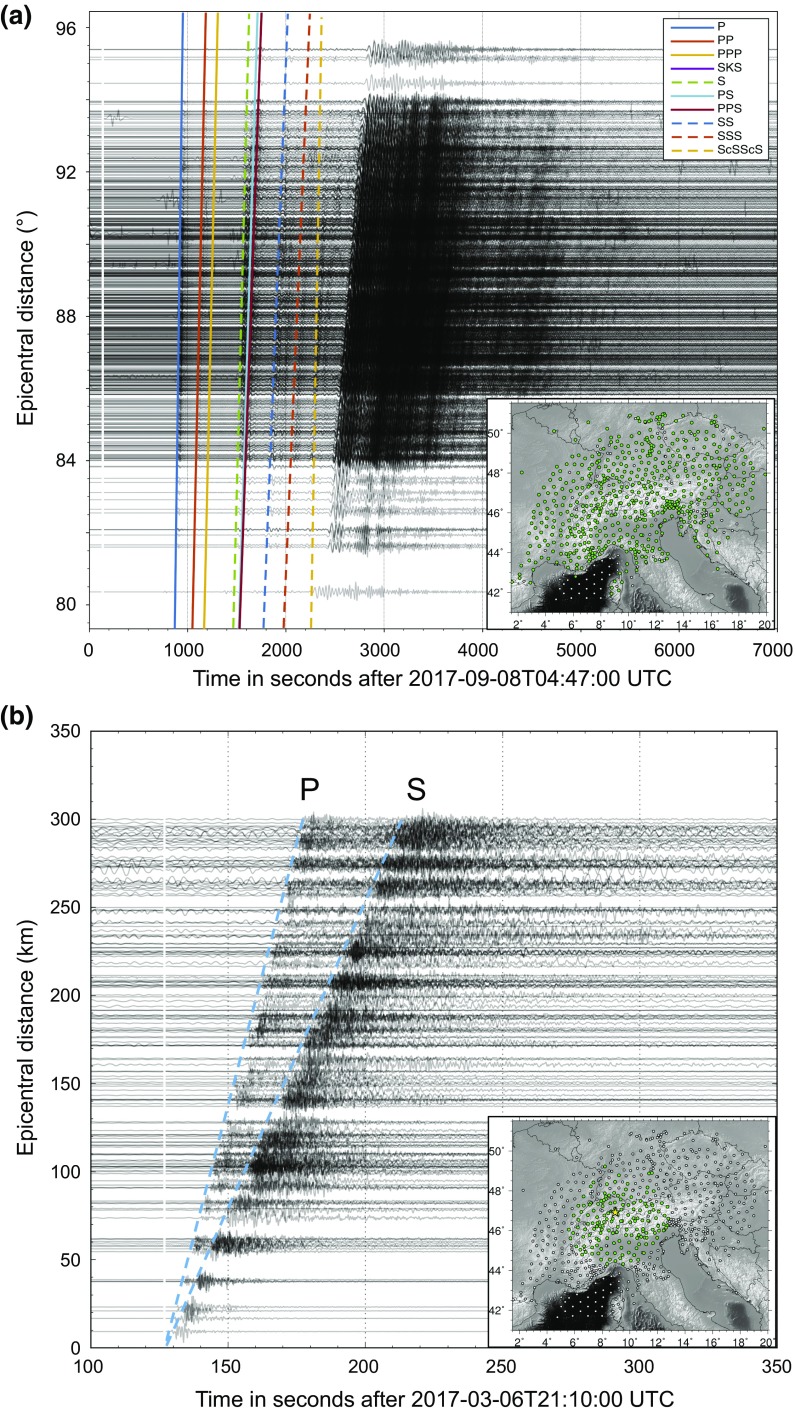
Waveform examples across the AASN. **a** Teleseismic waves following the 2017-09-08T04:49:19 (UTC) *M*_W_ 8.2 Mexico earthquake. Waveforms are band-pass filtered between 0.01 and 0.5 Hz. Theoretical teleseismic phase arrival times (see colour legend) are calculated by the Crazyseismic code (Yu et al. 2017). **b** Local and regional waves following the 2017-03-06T20:12:07 (UTC) *M*_L_ 4.6 Urnerboden (Switzerland earthquakes). Waveforms are band-pass filtered between 0.04 and 2 Hz. On both figures waveforms are shown for stations available for download on 20 March 2018, represented by green dots on the maps in the lower right corners. The white vertical line across the waveforms marks the origin time of the respective earthquake

To illustrate the power of the AASN, two examples of waveforms across the entire network are shown in Fig. 8, from a teleseismic and a local earthquake. In the teleseismic case, the coherency of waveforms across the array allows us to see body waves with multiple legs easily. In addition, two waveform animations from a teleseismic and a regional event are provided as Online Resource 5 and 6 to visualize the passage of waves through the AASN.

Over 18 GB of data is collected each day by the AASN, and the final size of the data archives is expected to be on the order of 15 TB. The seismological waveforms of the AASN are distributed through the European Integrated Data Archive (EIDA, http://145.23.252.222/eida/webdc3/), where the data from the 36 institutions that collect seismological data are archived across seven different nodes in five countries. Data from the temporary sites are available under the Z3 network code, while the virtual network “_ALPARRAY” comprises all stations of the AASN, permanent and temporary.

The AASN data are distributed with password protection. Data access is immediate for the Core Group of the AASN members. Registered seismological observatories with monitoring and alerting duties can use the Z3 data with real-time access and may report phase picks in their catalogues; however, waveforms shall not be published. The use of data for research by AlpArray participants requires a priori submission and approval of the research topic by the AASN Core Group.

The waveform data will be freely shared among the entire AlpArray Working Group at most 1 year after AASN operation has ended (currently planned for 1 January 2020). The waveform data will be freely available to the public 3 years after AASN operation has ended (currently planned for 1 January 2022).

The quality and completeness of AASN data have been, are and will be periodically reviewed and will be the scope of a forthcoming manuscript. For the earliest set of site noise characterization in different parts of the AASN, we refer to Fuchs et al. (2016), Molinari et al. (2016), Govoni et al. (2017) and Vecsey et al. (2017).

The AASN can be cited by referring to this paper and the temporary component (Z3) by the AlpArray Seismic Network (2015) seismic network DOI.

The AASN demonstrates the current capability of the community to integrate mobile data within infrastructures traditionally built for permanent network archives — which has been an ORFEUS focus for many years and has been funded through the EU projects NERIES, NERA and now EPOS-IP and SERA. More secure and professional management of the archives allows better curation and dissemination of these data sets and benefits the wider scientific community.

AASN data will be used for numerous seismological applications, which will be subject to forthcoming publications. The depth targets of these will range from shallow (e.g., sedimentary basins, landslides) to deep (e.g., mantle transition zone) depth. The methodologies expected to be applied include body and surface wave tomography, receiver functions, ambient-noise tomography, anisotropy studies, seismicity analysis, attenuation structure, various joint inversions and more. Some of the high-resolution tomography approaches employing body wave full waveform inversion expect to resolve length scales below 20 km. Thanks to its high spatial density and uniform coverage, the AASN is suitable for deep Earth imaging applications such as mapping ultra-low-velocity zones or reflectors near the core-mantle boundary, testing the large low-velocity anomaly beneath Africa or upper and mid-mantle seismic discontinuities in regions beneath India or the Atlantic Ocean.

## Conclusions and Perspective

A European geoscience initiative, coined AlpArray, has become a reality some 6 years after planning began in the summer of 2011. National funding from eight countries and the matching of national seismological instrument pools in time and space has enabled deployment of 276 temporary stations, including 30 ocean-bottom sensors, to fill spatial gaps between 352 permanent stations of the greater Alpine area across 11 countries. With a total of 628 stations, the AlpArray Seismic Network is now up and running, yielding unprecedented homogeneous and dense coverage to image orogenic structures and processes in 3D. We expect that the great effort invested in this broadband collaboration will not only be fruitful for basic and applied research (e.g., seismic hazard), but will also set a precedent for exemplary scientific cooperation across borders. Although ultimately successful, the diverse multinational funding streams had presented a real risk to this initiative. We expect that this experiment will be followed in the future by other groups of neighbouring countries to investigate their common geodynamic region and also by European observatories and research institutions to work jointly elsewhere. We therefore encourage funding agencies in Europe and elsewhere to think of mechanisms how proposal submission and funding decisions for such large-scale initiatives can be coordinated.

In the meanwhile, we expect a wealth of new images of local, regional and pan-Alpine structures to be revealed, as seen by seismological methods using the AASN and complementary experiments. These will foster the integration of other geophysical data (e.g., gravity) and the synthesis of numerous interpretative studies in the domains of geology and modelling, which ultimately lead the community to a much improved understanding of Alpine orogeny. Activities on specific research topics are coordinated inside thematic research groups (e.g., surface waves, ambient-noise and full waveform inversion; receiver functions; gravity; seismicity, seismotectonics and local earthquake tomography; seismic anisotropy), guaranteeing mutual collaboration and efficient updates on issues related to data quality, applied methodologies and scientific results.

## Electronic supplementary material

Below is the link to the electronic supplementary material.

Supplementary material 1 (MP4 6965 kb)

Supplementary material 2 (XLS 155 kb)

Supplementary material 3 (XLS 136 kb)

Supplementary material 4 (KMZ 26 kb)

Supplementary material 5 (MP4 14416 kb)

Supplementary material 6 (MP4 7994 kb)

Supplementary material 7 (DOCX 15 kb)
