# Supplementary material for: The AlpArray Seismic Network: A Large-Scale European Experiment to Image the Alpine Orogen
Source: Surv Geophys. 2018 Apr 18;39(5):1009–33. doi: 10.1007/s10712-018-9472-4 (PMC6428228; doi:10.1007/s10712-018-9472-4)
Supplement: Supplementary file 7 — Supplementary material 7 (DOCX 15 kb) [file 10712_2018_9472_MOESM7_ESM.docx]

**Supplementary material**

ESM_1.mp4: Animation showing the installation of temporary AlpArray stations (green) and the development of the permanent network (red) to construct the full AlpArray Seismic Network, between February 2015 and August 2017.

ESM_2.xlsx: Excel sheet of permanent stations and their properties, as of August 2017.

ESM_3.xlsx: Excel sheet of temporary stations and their properties, as of August 2017, with OBS closing dates added in March 2018.

ESM_4.kmz: GoogleEarth file with permanent and temporary stations and 250 km distance contour around the Alps

ESM_5.mp4: Teleseismic waves passing through the AASN following the Mw7.8 New Zealand earthquake on 13 November 2016. The signals are from the stations available near real-time on EIDA. Signals are filtered with a Butterworth band-pass filter between 50 s and 125 s, and normalised to between -1 and 1. The colour bar is reduced to between -0.1 and 0.1 to enhance the amplitude variations in the animation. The bottom waveforms highlight two stations, contoured on the map with respective colours of the cursor. All data were processed with softwares python and ObsPy (Beyreuther et al. 2010; Megies et al. 2011; Krischer et al. 2015).

ESM_6.mp4: Regional and local waves passing through the AASN following the Mw6.5 Norcia, Italy earthquake on 30 October 2016. The signals are from the stations available near real-time on EIDA. Signals are filtered with a Butterworth band-pass filter between 12 s and 100 s, and normalised to between -1 and 1. The colour bar is reduced to between -0.1 and 0.1 to enhance the amplitude variations in the animation. The bottom waveforms highlight two stations, contoured on the map with respective colours of the cursor. All data were processed with softwares python and ObsPy (Beyreuther et al. 2010; Megies et al. 2011; Krischer et al. 2015).
